# Medical Image Segmentation Based on a Hybrid Region-Based Active Contour Model

**DOI:** 10.1155/2014/890725

**Published:** 2014-06-16

**Authors:** Tingting Liu, Haiyong Xu, Wei Jin, Zhen Liu, Yiming Zhao, Wenzhe Tian

**Affiliations:** ^1^College of Information Science and Engineering, Ningbo University, Ningbo 315211, China; ^2^College of Science & Technology, Ningbo University, Ningbo 315211, China

## Abstract

A novel hybrid region-based active contour model is presented to segment medical images with intensity inhomogeneity. The energy functional for the proposed model consists of three weighted terms: global term, local term, and regularization term. The total energy is incorporated into a level set formulation with a level set regularization term, from which a curve evolution equation is derived for energy minimization. Experiments on some synthetic and real images demonstrate that our model is more efficient compared with the localizing region-based active contours (LRBAC) method, proposed by Lankton, and more robust compared with the Chan-Vese (C-V) active contour model.

## 1. Introduction

Medical images are generally ambiguous. If objects of interest and their boundaries can be located correctly, meaningful visual information would be provided to the physicians, making the following analysis much easier. Within the numerous image segmentation algorithms, active contour model is widely used with its clear curve for the object.

According to the curve representation, there are two main kinds of active contour models: parametric models and geometric models. Parametric active contour models use parameterized curves to represent the contours. Snake model, proposed by Kass et al. in [1], is a representative and popular one among parametric active contour models. The model requires a constant curve to detect the boundary of the image. In early age, the parametric active contour model is an efficient framework for biometric image segmentation. However, it cannot represent the topology changes such as the merging and splitting of the evolving curve [1].

The geometric active contour model, combining level set method and curve evolution theory, allows cusps, corners, and automatic topological changes. It can solve problems of curve evolution in the parametric active contour model and extend the application region of the active contour model.

Considering the parametric/geometric active contour model propagating toward a local optimum and thus exhibiting sensitivity to initial conditions, Bresson et al. proposed a new global optimization method in [2]. This fast active contour is based on the level set method, replacing the framework with convex relaxation approaches. Therefore, the model does not rely on the initial information with speed.

According to the energy, there are two main categories of active contour models: edge-based models [1–6] and region-based models [7–24]. Edge-based active contour models rely on the image gradient to stop the evolving contours on the desired object boundaries [6]. For images with weak boundaries, the energy functional of the edge-based active contour models will hardly approach zero on the boundaries of the objects and the evolving curve may pass through the true boundaries. Therefore, the edge-based active contour models always fail to segment medical images properly, as blur or weak edge usually occur in the medical images, especially in MRI brain images, which typically contain large area of blur boundaries between gray matter and white matter [12]. Compared with the edge-based active contour models, the region-based active contour models do not utilize the image gradient; they utilize image statistics inside and outside the contours to control the evolution with better performance for images of weak edges or without edges. Many region-based active contour algorithms are based on the assumption that an image can be approximated by global intensity. For example, Chan and Vese proposed a famous Chan-Vese (CV) model in [7] and Yezzi et al. proposed a fully global approach in [16], deriving a set of coupled curve evolution equations from a single global cost functional to promote multiple contours to segment multiple-region image.

CV model, also known as PC (piecewise constant) model, proposed in [7], is a simplified Mumford-Shah function. The model utilizes the global mean intensities of the interior and exterior regions of images. Thus, it has good segmentation result for the objects with weak or discrete boundaries but often has erroneous segmentation for images with intensity inhomogeneity. However, due to technical limitations or artifacts introduced by the object being imaged, intensity inhomogeneity often occurs in many medical images [12, 13, 25].

Many implementation schemes have been proposed to break the restrictions of CV model. For example, in [8, 9], two similar region-based models are proposed independently. These models are based on a general piecewise smooth (PS) formulation which is originally proposed by Mumford and Shah in [26] and have been known as piecewise smooth (PS) models. The PS models can handle segmentation problems which are caused by intensity inhomogeneity. Lankton and Tannenbaum proposed localizing region-based active contours (LRBAC) in [15], allowing any region-based segmentation energy to be reformulated in a local way. The technique they proposed can be used with any global region-based active contour energy, segmenting objects with heterogeneous statistics. However, they are computationally too expensive. One way to reduce the computational cost being proposed in [9] is to use a contour near the object boundaries as the initial contour.

In [11, 17, 18, 23, 27–30], local region-based active contour models are proposed to overcome the difficulty caused by intensity inhomogeneity. The local binary fitting (LBF) model in [10] and the region-scalable fitting (RSF) model in [11] being proposed by Li et al. are the most popular models. LBF model utilizes image information in local regions. RSF model draws upon intensity information in local regions at a controllable scale. These two models have similar capability to handle intensity inhomogeneity. However, they are also sensitive to initialization.

To make the segmentation efficient, Piovano et al. [25] used convolutions to quickly compute localized statistics and yield results similar to piecewise smooth segmentation. A model proposed in [13] is to deal with spatial perturbations of the image intensity directly. In [14], Lankton et al. proposed a similar flow based on computing geodesic curve in the space of localized means rather than approximating a piecewise smooth model. The technique can identify object boundaries accurately and reduce dependence on initial curve placement.

More recently, Xu et al. proposed a hybrid active contour in [31]. The model incorporates the GAC model, which is an edge-based active contour model, and the CV model, which is a region-based active contour model. The new model was called as geodesic intensity fitting (GIF) model. It was then extended to two models: global geodesic intensity fitting (GGIF) model and local geodesic intensity fitting (LGIF) model. The GGIF model is for images with intensity homogeneity. And the LGIF model is for images with intensity inhomogeneity.

Motivated by the work in [31], we plan to propose a model which is based on the region information of the images. While it is fast but not accurate in using global information and it is accurate but not fast in using local information, the new function will use both of global and local information to attain the correct result quickly. Inspired by [7, 15], a hybrid region-based active contour model is presented for image segmentation in this paper. The global information is provided by CV model. The local information is described by applying the framework proposed in [15] to the energy in [16], localizing the energy. The weights between the local and global fitting terms are applied to avoid computationally expensive and erroneous segmentation.

## 2. Background

### 2.1. Chan-Vese Model

The Chan-Vese (CV) model [7] is a special case of the Mumford-Shah problem [26]. Given the curve *C* = ∂*ω*, with *ω* ⊂ Ω being an open subset, for the image *I*(*x*, *y*) on the image domain Ω, the energy functional they proposed is

(1) F(c1,c2,C)=λ1∫inside(C)|I(x,y)−c1|2dx dy+λ2∫outside(C)|I(x,y)−c2|2dx dy+μ|C|,

where inside(*C*) and outside(*C*) represent the regions outside and inside the contour *C*, respectively. The constants *c*_1_ and *c*_2_ are the intensity averages of inside(*C*) and outside(*C*), respectively. |*C*| is the length of the contour *C*, the third term in the right hand side of (1), which is introduced to regularize the contour *C*. The parameters *μ*, *λ*_1_, and *λ*_2_ are positive constants, usually fixing *λ*_1_ = *λ*_1_ = 1.

To solve the minimization problem, the level set method proposed in [32] is used in which the unknown curve *C* is replaced by the unknown level set function *ϕ*(*x*, *y*), considering that *ϕ*(*x*, *y*) > 0 if the point (*x*, *y*) is inside *C*, *ϕ*(*x*, *y*) < 0 if the point (*x*, *y*) is outside *C*, and *ϕ*(*x*, *y*) = 0 if the point (*x*, *y*) is on *C*. Thus, the energy functional *F*(*c*_1_, *c*_2_, *C*) can be reformulated in terms of the level set function *ϕ*(*x*, *y*) as follows:

(2) F(c1,c2,ϕ) =λ1∫Ω|I(x,y)−c1|2Hε(ϕ(x,y))dx dy  +λ2∫Ω|I(x,y)−c2|2(1−Hε(ϕ(x,y)))dx dy  +μ∫Ωδε(ϕ(x,y))|∇ϕ(x,y)|dx dy,

where *H*_*ε*_(*Z*) and *δ*_*ε*_(*Z*) are, respectively, the regularized approximation of Heaviside function *H* and delta function *δ* as follows:

(3) $$\begin{array}{c} H\left(z\right)=\{\begin{array}{cc} 1, & \text{if}\;z\geq 0\\ 0, & \text{if}\;z<0, \end{array}\;\delta \left(z\right)=\frac{d}{d_{z}}H\left(z\right). \end{array}$$

Using the Euler-Lagrange equations to solve the minimization problem of (2), the level set function *ϕ*(*x*, *y*) can be updated by the following gradient descent method:

(4) $$\begin{array}{c} \frac{\partial \phi }{\partial t}=\delta_{\varepsilon }\left(\phi \right)\left[\mu \mathrm{div}\left(\frac{\nabla \phi }{\left|\nabla \phi \right|}\right)-\lambda_{1}{\left(I-c_{1}\right)}^{2}+\lambda_{2}{\left(I-c_{2}\right)}^{2}\right], \end{array}$$

where *c*_1_ and *c*_2_ can be expressed, respectively, as follows:

(5) c1(ϕ)=∫ΩI(x,y)Hε(ϕ(x,y))dx dy∫ΩHε(ϕ(x,y))dx dy,c2(ϕ)=∫ΩI(x,y)(1−Hε(ϕ(x,y)))dx dy∫Ω(1−Hε(ϕ(x,y)))dx dy.

Compared with other active contour models, CV model is far less sensitive to the initialization. The initial curve can be placed anywhere in the image, and it can detect both contours with or without gradient. However, as the model uses global information of the image, the optimal constants *c*_1_ and *c*_2_ will not be accurate if the image intensities in inside(*C*) and outside(*C*) are not homogeneous. Thus, the CV model generally fails to segment images with intensity inhomogeneity.

### 2.2. Coupled Curve Evolution Equations

Yezzi et al. proposed a fully global approach to image segmentation via coupled curve evolution equations in [16]. Followed by [15], we call it mean separation (MS) energy as it uses mean intensities. The technique can “pull apart” the values of two or more image statistics and is useful for segmenting images of a known number of region types. The approach can promote multiple contours simultaneously toward the region boundaries. In this paper, we only refer to the simple case of bimodal imagery in which there are two types of regions, foreground and background.

Given a binary image *I*(*x*, *y*) on the image domain Ω, foreground region *R* of intensity *I* ^*r*^, and background region *R* ^*c*^ of intensity *I* ^*c*^, *I* ^*r*^ ≠ *I* ^*c*^. Initial closed curve $\vec{C}$ encloses some portions of *R* and some portions of *R* ^*c*^. The mean intensities *u* and *v* inside and outside the curve, respectively, are bounded above and below by *I* ^*r*^ and *I* ^*c*^; when $\vec{C}=\partial R$, an upper bound of |*I* ^*r*^ − *I* ^*c*^| is uniquely attained. That means that foreground and background regions should have maximal separate mean intensities. The energy is

(6) $$\begin{array}{c} E=-\frac{1}{2}{\left(u-v\right)}^{2}. \end{array}$$

The gradient of *u* and *v* is

(7) $$\begin{array}{c} \nabla u=\frac{I-u}{A_{u}}\vec{N},\\ \nabla v=-\frac{I-v}{A_{v}}\vec{N}, \end{array}$$

where *A*_*u*_ and *A*_*v*_ denote the area of interior and exterior of $\vec{C}$, respectively. $\vec{N}$ denotes the outward unit normal of $\vec{C}$, which will become $-\vec{N}$ with respect to the exterior of  $\vec{C}$. The gradient flow of *E* is

(8) $$\begin{array}{c} \frac{d\vec{C}}{dt}=-\nabla E=\left(u-v\right)\left(\frac{I-u}{A_{u}}+\frac{I-v}{A_{v}}\right)\vec{N}. \end{array}$$

To counter the effect of noise, a penalty on the arc length of the curve is added to functional (6):

(9) $$\begin{array}{c} E=-\frac{1}{2}{\left(u-v\right)}^{2}+\alpha \int_{\vec{C}}ds. \end{array}$$

The penalty regularizes the gradient flow as

(10) $$\begin{array}{c} \frac{d\vec{C}}{dt}=\left(u-v\right)\left(\frac{I-u}{A_{u}}+\frac{I-v}{A_{v}}\right)\vec{N}-\alpha k\vec{N}. \end{array}$$

Equation (9) is always expressed in a more general way:

(11) $$\begin{array}{c} E=-\frac{1}{2}{||u-v||}^{2}+\alpha \int_{\vec{C}}ds. \end{array}$$

In contrast to other region-based snake algorithms, the technique requires no prior knowledge of evolution, exhibiting more robustness to initial contour placement and noise. However, the method is not suitable for the heterogeneous objects as they use the global statistics.

## 3. The Proposed Method

In this section, we propose a hybrid region-based active contour model which can segment images with intensity inhomogeneity accurately and efficiently. As mentioned in Section 2, global region-based active contour models can segment images with weak boundaries but are not suitable for images with intensity inhomogeneity. For this kind of images, we can use local region-based active contour models to do the segmentation. However, using local information will cause high computation cost. The energy functional *E* ^Hybrid^ can share the local and global advantages without adding extra computation when compared to pure global schemes. The energy functional is defined as

(12) $$\begin{array}{c} E^{\text{Hybrid}}=\alpha E^{\text{Global}}+\beta E^{\text{Local}}, \end{array}$$

where *α* and *β* are positive parameters that control the contribution of the global and local energy. As lots of images contain noises, the contour may tend to weave around or encircle extremely small regions due to noise. To counter such effect and keep the curve smooth, we add a regularization term *L*(*ϕ*) as is commonly done. The term is defined related to the arc length of the contour *C* during evolution. The final energy is given as follows:

(13) $$\begin{array}{c} E^{\text{Hybrid}}=\alpha E^{\text{Global}}+\beta E^{\text{Local}}+\omega L\left(\phi \right). \end{array}$$

### 3.1. Global Energy

Let Ω ⊂ *R* ^2^ be the image domain; let *I* : Ω → *R* be a given gray level image; the global energy we use is

(14) $$\begin{array}{c} E^{\text{Global}}=\int_{\text{inside}(C)}{\left|I(y)-m\right|}^{2}dy+\int_{\text{outsid}(C)}{\left|I(y)-n\right|}^{2}dy, \end{array}$$

where *m* and *n* are the mean intensity of foreground and background of the image, respectively. According to the level set method, *C* ⊂ Ω is represented by the zero level set function *ϕ* : Ω → *R*, such that

(15) $$\begin{array}{c} C=\partial \omega =\left\{\left(x,y\right)\in \Omega :\phi \left(x,y\right)=0\right\},\\ \text{inside}\left(C\right)=\omega =\left\{\left(x,y\right)\in \Omega :\phi \left(x,y\right)>0\right\},\\ \text{outside}\left(C\right)=\Omega \setminus \varpi =\left\{\left(x,y\right)\in \Omega :\phi \left(x,y\right)<0\right\}. \end{array}$$

Using the Heaviside function *H*, (14) can be rewritten as

(16) EGlobal=∫Ωy(Hϕ(y)(I(y)−m)2+(1−Hϕ(y))(I(y)−n)2)dy.

In practice, the Heaviside function *H* is approximated by a smooth function *H*_*ε*_ defined by

(17) $$\begin{array}{c} H_{\varepsilon }\left(z\right)=\frac{1}{2}\left[1+\frac{2}{\pi }\arctan\left(\frac{z}{\varepsilon }\right)\right]. \end{array}$$

The derivative of *H*_*ε*_ is *δ*_*ε*_:

(18) $$\begin{array}{c} \delta_{\varepsilon }\left(Z\right)=H_{\varepsilon }^{\prime }\left(z\right)=\frac{1}{\pi }\frac{\varepsilon }{\varepsilon^{2}+z^{2}}, \end{array}$$

where *ε* is a positive constant.

### 3.2. Local Energy

As mentioned in Section 2, the local energy can handle segmentation of images with intensity inhomogeneity. Inspired by [15], we use a ball function *B* to mask local regions. The local energy functional is

(19) ELocal=∫Ωx∫ΩyB(x,y)FLocaldx dy=∫Ωx∫ΩyB(x,y)(ux−vx)2dx dy,

where *B*(*x*, *y*) is a ball function, centered at *x*, and can be expressed as

(20) $$\begin{array}{c} B\left(x,y\right)=\{\begin{array}{cc} 1, & ||x-y||<r\\ 0, & \text{otherwise}, \end{array} \end{array}$$

where *r* is the ball radius. The function will be 1 when the point *y* is within a ball and 0 otherwise.

*u*_*x*_ and *v*_*x*_ are the mean values of the intensity inside and outside the contour in the local ball region (centered at *x*). The expressions of *u*_*x*_ and *v*_*x*_ are as follows:

(21) $$\begin{array}{c} u_{x}=\frac{\int_{\Omega_{y}}B\left(x,y\right)H\phi \left(y\right)I\left(y\right)dy}{\int_{\Omega_{y}}B\left(x,y\right)H\phi \left(y\right)dy},\\ v_{x}=\frac{\int_{\Omega_{y}}B\left(x,y\right)\left(1-H\phi \left(y\right)\right)I\left(y\right)dy}{\int_{\Omega_{y}}B\left(x,y\right)\left(1-H\phi \left(y\right)\right)dy}. \end{array}$$

To get the optimum result, *u*_*x*_ and *v*_*x*_ should be very different at every *x* along the contour. That means local foreground and background should be different rather than constant.

### 3.3. Total Energy Formulation

For lots of images containing noises, the contour may tend to weave around or encircle extremely small regions due to noise. To offset such effect and keep the curve smooth, we add a regularization term as is commonly done. The term is defined related to the arc length of the contour *C* during evolution:

(22) $$\begin{array}{c} L\left(\phi \right)=\int_{\Omega_{x}}\delta \phi \left(x\right)\left|\nabla \phi \left(x\right)\right|dx, \end{array}$$

where *δ*_*ε*_ is the derivative of *H*_*ε*_:

(23) $$\begin{array}{c} \delta_{\varepsilon }\left(Z\right)=H_{\varepsilon }^{\prime }\left(z\right)=\frac{1}{\pi }\frac{\varepsilon }{\varepsilon^{2}+z^{2}}. \end{array}$$

The energy functional in (13) can be rewritten as

(24) EHybrid(ϕ,m,n,ux,ux) =α∫Ωy(Hϕ(y)(I(y)−m)2     +(1−Hϕ(y))(I(y)−n)2)dx dy  +β∫Ωx∫ΩyB(x,y)(ux−vx)2dx dy  +ω∫Ωxδϕ(x)|∇ϕ(x)|dx.

By applying the standard gradient descent method, the constants *m* and *n*, optimal means *u*_*x*_ and *v*_*x*_, and level set function *ϕ* which minimize the energy functional (24) are obtained by

(25) ∂ϕ∂t(x)=αδϕ(y)[−(I−m)2+(I−n)2]+β∫ΩyB(x,y)δϕ(y)   ×((I(y)−ux)2Au−(I(y)−vx)2Av)dy+ωδϕ(x)div⁡(∇ϕ(x)|∇φ(x)|)=αδϕ(y)[−(m−n)(2I−m−n)]+β∫ΩyB(x,y)δϕ(y)   ×((I(y)−ux)2Au−(I(y)−vx)2Av)dy+ωδϕ(x)div⁡(∇ϕ(x)|∇ϕ(x)|),

where *A*_*u*_ is the area of local interior *A*_*u*_ and *A*_*v*_ is the area of local exterior:

(26) $$\begin{array}{c} A_{u}=\int_{\Omega_{y}}B\left(x,y\right)H\varphi \left(y\right)dy\\ A_{v}=\int_{\Omega_{y}}B\left(x,y\right)\left(1-H\varphi \left(y\right)\right)dy. \end{array}$$

## 4. Implementation and Experimental Results

The level set evolution equation (25) is implemented using a simple finite differencing. The temporal partial derivative ∂*ϕ*/∂*t* is discretized as the forward difference. The approximation of (25) can be simply written as

(27) $$\begin{array}{c} \phi_{ij}^{k+1}=\phi_{ij}^{k}+\Delta tL\left(\phi_{ij}^{k}\right), \end{array}$$

where *L*(*ϕ*_*ij*_^*k*^) is the approximation of the right hand side in (25).

In our implementation, the level set function *ϕ* can be simply initialized as

(28) $$\begin{array}{c} \phi^{0}=\{\begin{array}{cc} -c_{0}, & x\in \Omega_{0}-\partial \Omega \\ 0, & x\in \partial \Omega \\ c_{0}, & x\in \Omega -\Omega_{0}, \end{array} \end{array}$$

where *C*_0_ > 0 is a constant, Ω_0_ is the subset of the image domain Ω, and ∂Ω is the boundary of Ω_0_. We test the proposed method with some synthetic and natural images from different modalities. Experiments are implemented on a computer with Intel Core 2 duo, 2.53 GHZ CPU, 2.0 GB RAM, Windows 7 ultimate, and MATLAB 7.12. Unless otherwise specified, we use the following default settings of the parameters: time step Δ*t* = 0.45 and *ω* = 0.1. The parameters *α* and *β* vary from 0 to 2 according to the degree of inhomogeneity, *α* ≈ *β*.

### 4.1. Synthetic Imagery

**Figure 1:**
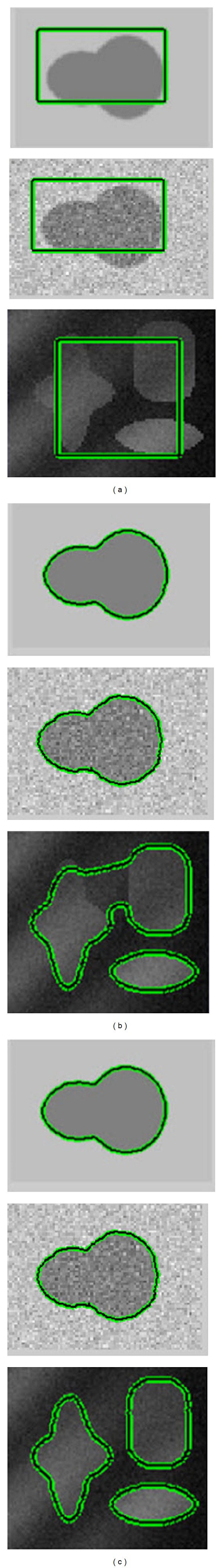
Comparison of our method with the LRBAC method. The initial contours and the final contours are plotted as the green contours. (a) Initial contours; (b) results of the LRBAC method; and (c) results of our method.

Figure 1 shows experimental results of both LRBAC [15], which used only local information of the image, and the proposed method for three images. Among that, noisy gourd and three objects are with noises. The original images with the same initial contour are listed in Figure 1(a). While the segmentation results of LRBAC method and the proposed method are shown Figures 1(b) and 1(c), respectively. We observed that both LRBAC method and the proposed method could well segment the image of gourd (59∗67) and the image of noisy gourd (59∗67). For the image of three objects (79∗75), LRBAC method gets trapped into a local minimum without taking global image information into account while our model extracts the object boundary successfully. As both LRBAC method and the proposed method use the regularization term, contours are kept smoothly under the noisy condition. Otherwise, noises would be recognized as objects.

For the image of gourd and the image of noisy gourd, although both LRBAC method and our method can segment them well, our method takes far less time to get the satisfied result, being more efficient than the LRBAC method. Iterations and CPU time are listed in Table 1.

**Figure 2:**
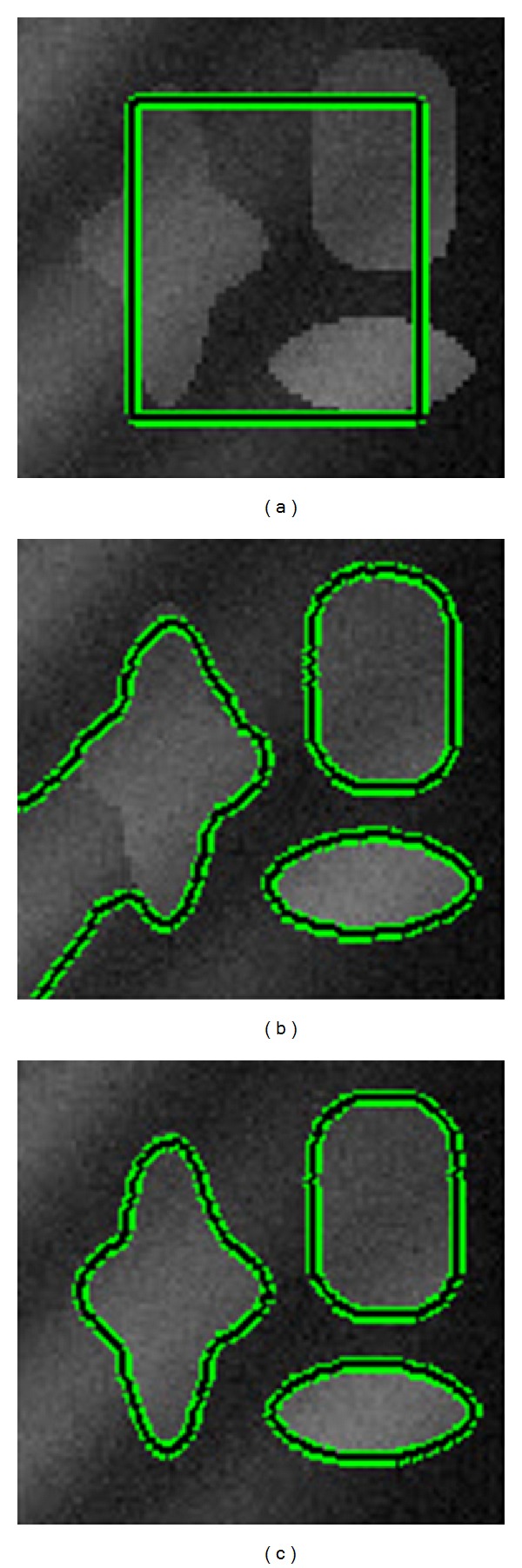
Comparison of our method with the CV model. (a) Initial contours; (b) results of the CV model; and (c) results of our method.

Figure 2 shows experimental results of both CV method and the proposed method on the image of three objects (79∗75). The image was created with intensity inhomogeneity and contaminated by Gaussian noise.  Table 2 shows Iterations and CPU time of these two methods. We can notice that although CV model spends less time in curve evolution, it leaks at the boundary of object and cannot segment the object accurately.

### 4.2. Real Imagery

**Figure 3:**
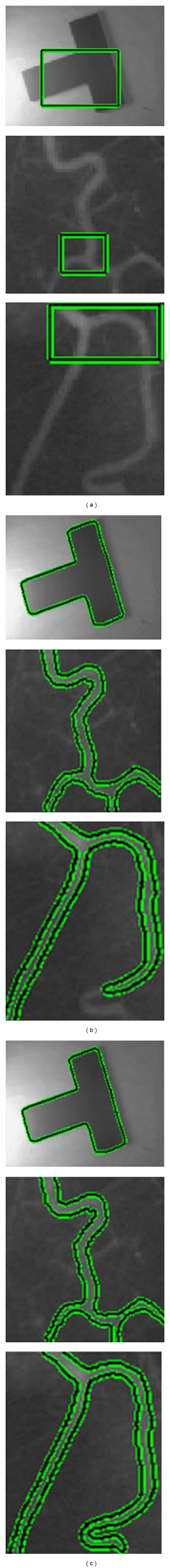
Comparison of our method with the LRBAC method. (a) Initial contours; (b) results of the LRBAC method; and (c) results of our method.

Intensity inhomogeneity often occurs in real images.  Figure 3 shows a real image of a T-shaped object (96∗127) and two X-ray images of vessel (110∗111 and 131∗103) with intensity inhomogeneity. T-shaped object with intensity inhomogeneity is due to nonuniform illumination. For X-ray images of vessel, some parts of the boundaries are quite weak and some parts of the background intensities are even higher than the vessel, which makes it a nontrivial task to segment the image with global method. We compare the segmentation ability of our method with the LRBAC method. As shown in Figure 3 and Table 3, for image of Vessel 1, our method gains faster result than the LRBAC method and, for image of Vessel 2, our method gains better results than the LRBAC method.

**Figure 4:**
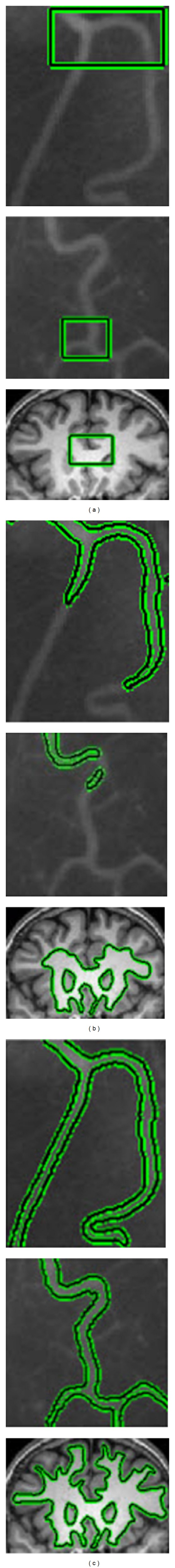
Comparison of our method with the CV model. (a) Initial contours; (b) results of the CV model; and (c) results of our method.

Figure 4 shows experimental results of both CV model and our method on four typical medical images with intensity inhomogeneity: two X-ray images of vessels (131∗103 and 110∗111) and one brain MR image (121∗81). From Figure 4, we can see the brain MR image has some intensities of white matter in the upper part, which are even lower than those of the gray matter in the lower part. The experiment results show that even CV model can drive curve faster than our method, which fails to extract the object boundaries (Table 4).

## 5. Conclusion

In this paper, we propose a hybrid region-based active contour model for image segmentation. The proposed method can improve the ability of the CV model to deal with intensity inhomogeneity. Meanwhile, it makes segmentation more efficient compared to the LRBAC method. Experimental results on both synthetic and real images demonstrate that the proposed method can handle both better intensity inhomogeneity and robustness to noise compared with CV model and the LRBAC method.

It should be noted that the energy functional for our model is nonconvex and therefore has local minima, which makes our model sensitive to the initialization of the contour. Future work can be made to apply proper algorithms to develop globally optimal active contours.

## Figures and Tables

**Table 1:** Iterations and CPU time for the LRBAC method and our method for images in Figure 1.

	Gourd		Noisy gourd		Three objects	
	Iterations	Time (s)	Iterations	Time (s)	Iterations	Time (s)
LRBAC method	200	2.822	360	5.717	1200	27.612

Our method	60	0.894	160	2.641	2200	55.816

**Table 2:** Iterations and CPU time for CV model and our method for images in Figure 2.

	Three objects	
	Iterations	Time (s)
CV model	800	4.774

Our method	2200	55.816

**Table 3:** Iterations and CPU time for the LRBAC method and our method for images in Figure 3.

	T-shape		Vessel 1		Vessel 2	
	Iterations	Time (s)	Iterations	Time (s)	Iterations	Time (s)
LRBAC method	1220	36.678	480	17.156	2500	126.700

Our method	760	23.648	380	13.516	2500	141.290

**Table 4:** Iterations and CPU time for CV model and our method for images in Figure 4.

	Vessel 1		Vessel 2		Brain	
	Iterations	Time (s)	Iterations	Time (s)	Iterations	Time (s)
CV model	880	7.003	680	4.705	3300	21.913

Our method	2500	141.290	380	13.516	1080	47.928
